# Knowledge, Awareness, and Attitude of Physicians Toward Child Abuse: A Cross-Sectional Study

**DOI:** 10.7759/cureus.60420

**Published:** 2024-05-16

**Authors:** Dunya N AlFaraj, Hussain J Aljubran, Omar A Bamalan, Randa M Dibo, Feras K Mimarji, Salem A AlMarri, Maryam A Alfaraj, Fatmah S Almahroos, Tasneem S Almahroos

**Affiliations:** 1 Department of Emergency Medicine, King Fahd Hospital of the University, Imam Abdulrahman Bin Faisal University, Dammam, SAU; 2 Department of Pediatrics, King Fahd Hospital of the University, Imam Abdulrahman Bin Faisal University, Dammam, SAU; 3 Department of Emergency Medicine, Johns Hopkins Aramco Healthcare, Dhahran, SAU; 4 Department of Emergency Medicine, King Fahad Hospital, Hofuf, SAU; 5 Department of Pediatrics, Eastern Health Cluster, Dammam, SAU

**Keywords:** attitude, knowledge, awareness, physicians, non-accidental injury, child abuse

## Abstract

Introduction: Child abuse refers to any type of mistreatment of a child, perpetrated by a parent, caregiver, or another individual in a custodial capacity, which may lead to instances of physical, sexual, or emotional abuse. Physicians play a crucial role in identifying and managing this phenomenon in the healthcare setting, as the number of unreported cases increases globally.

Methods: A questionnaire-based cross-sectional study was conducted between 2022 and 2023 among physicians practicing in Saudi Arabia to assess their knowledge, awareness, and attitude toward child abuse. The data were analyzed using IBM SPSS Statistics for Windows, Version 26 (Released 2019; IBM Corp., Armonk, New York, United States).

Results: A total of 153 physicians were involved in this study, in which 65 participants (42.5%) indicated poor awareness of child abuse, while 79 participants (51.6%) indicated moderate knowledge of child abuse. Additionally, lack of knowledge was the most common barrier to reporting child abuse in 87 participants (56.9%). A positive significant correlation was identified between awareness and knowledge and between knowledge and attitude. Also, it was found that a higher attitude score was more associated with being male, having less experience, practicing in the emergency medicine department, and working in a governmental hospital.

Conclusion: These results highlight the significance of implementing specialized training programs and workshops focused on identifying and reporting child abuse, as well as providing guidelines for recognizing signs of abuse and taking appropriate intervention measures.

## Introduction

Child abuse refers to any type of mistreatment of a child, perpetrated by a parent, caregiver, or another individual in a custodial capacity, which may lead to instances of physical, sexual, or emotional abuse [1]. Globally, the reported estimates of the prevalence of child abuse vary significantly, ranging from almost 0% to over 90% [2,3]. The differences in prevalence rates may stem from actual variations in the incidence of child maltreatment, such as variations in the types of maltreatment, gender disparities, or the diverse geographical backgrounds of the samples. Thus, this phenomenon is a serious but often overlooked and disregarded issue impacting children globally. A recent study that was published in the United States reported that 9.2% of children experienced abuse [4]. In Saudi Arabia, the first documented case was not published in medical literature until 1990, highlighting the previously limited recognition of this problem [5]. However, the recent extent of this issue in Saudi Arabia remained unknown because of the absence of precise data on the frequency and prevalence of child abuse, despite hospitals experiencing a rise in reported cases. Child abuse is a significant concern, as the growing number of unreported cases amplifies the potential consequences, which may ultimately result in death. A previous study that was published in North Carolina found that 60% of deaths caused by child abuse were unreported, underscoring the significance of this issue [6].

Physicians and other healthcare staff have ethical, moral, and legal responsibilities pertaining to the identification and management of child abuse cases. Physicians play a crucial role in identifying and preventing child abuse within healthcare settings, as they are usually the first to encounter and report such cases [7]. Considering the role of physicians as the first medical contact point in the diagnosis and prevention of this phenomenon, this study aims to assess the Saudi physicians’ knowledge (information, understanding, and insight into the causes, risk factors, consequences, and available resources related to child abuse), awareness (beliefs and feelings of the existence, nature, and impact of child abuse), and attitude (behavioral tendencies regarding child abuse) on child abuse and to evaluate the quality of education given to them.

## Materials and methods

Study design and participants

A questionnaire-based cross-sectional study was conducted among physicians practicing in Saudi Arabia at King Fahd University Hospital, Khobar. All physicians in any specialty who are practicing in Saudi Arabia and encountering pediatric patients during their practice were invited to participate, including males and females of any age. Those who did not complete the survey were excluded from the study. The study was approved by the Institutional Review Board of Imam Abdulrahman Bin Faisal University (IRB number: 2021-01-429), and participants gave their informed consent to be involved.

Data collection tools and processes

Physicians were recruited using a convenience sampling method and were asked to participate by filling out an online questionnaire. The survey was conducted using QuestionPro, a questionnaire software based in Seattle, United States. The questionnaire was adapted from two previous studies that measured the physicians’ knowledge, awareness, and attitude toward child abuse and was modified according to our aim [8,9]. Additionally, to accurately measure the knowledge of physicians, cases with pictures were added from the Atlas of Pediatric Emergency Medicine book [10]. A pilot study was conducted using an adjusted questionnaire with 10 physicians. The aim was to assess the time needed to complete the questionnaire and the clarity of the questions, with adjustments made as necessary. The online-based questionnaire was initiated in December 2022 and remained accessible for four months. On average, it took participants eight minutes to complete the survey. To avoid duplicate responses, participants are permitted to submit their responses via the provided link only once.

Questionnaire criteria

Physicians' awareness toward child abuse has been assessed using a five-item questionnaire, with the correct answer for each question identified and coded with 1 while the incorrect answer was coded with 0. The total awareness score was calculated by adding all five items. Scores ranging from 0 to 5 points were achieved. The higher the score, the higher the awareness about child abuse. Respondents were classified as having poor awareness if the score was from 0 to 2 points, 3 points were considered moderate, and 4 to 5 points were considered as good awareness levels.

The attitude toward child abuse has been assessed using a three-item questionnaire with a five-point Likert scale category ranging from "strongly disagree" coded with 1 to "strongly agree" coded with 5 as answer options. The involved questions were: "I feel that it is essential to have educational programs related to child abuse for healthcare providers", "I feel that reporting cases of suspected child abuse is a responsibility of training physicians," and "I feel confident in reporting in diagnosing and treating children with suspected child abuse." The total attitude has been calculated by adding all three questions. A score ranging from 3 to 15 points has been generated. The higher the score, the higher the attitude toward child abuse. By using 50% and 75% as cutoff points to determine the level of attitude, physicians were considered negative if the score was less than 50%; 50% and 75% were considered neutral, and above 75% were considered positive attitude levels.

The knowledge about recognizing child abuse based on radiographs of different cases has been assessed using a 16-item questionnaire, with the correct answer for each question identified and coded with 1 while the incorrect answer was coded with 0. The total knowledge score has been calculated by adding all 16 items. The higher the score, the higher the knowledge about different case scenarios. Similar criteria taken from attitude questionnaires were applied to determine the level of knowledge (cutoff points of 50% and 75% from the total score).

Statistical analysis

The data were analyzed using the software program IBM SPSS Statistics for Windows, Version 26 (Released 2019; IBM Corp., Armonk, New York, United States). Descriptive statistics were given as numbers and percentages (%) for all categorical variables, while continuous variables were calculated and summarized as mean and standard deviation. The differences in the awareness, attitude, and knowledge scores in relation to physicians' socio-demographic characteristics were evaluated using the Mann-Whitney Z-test and Kruskal Wallis H-test. The normality test (statistical collinearity) was performed using the Shapiro-Wilk test as well as the Kolmogorov-Smirnov test. The results show that awareness, attitude, and knowledge scores follow a non-normal distribution. Thus, the non-parametric test was applied. Also, the Spearman correlation coefficient was performed to determine the correlation between the awareness, attitude, and knowledge scores. Values were considered significant with a p-value of less than 0.05.

## Results

This cross-sectional study involved 153 physicians (response rate: 72%). As seen in Table 1, 63 participants (41.2%) were aged between 30 and 39 years old, with 91 physicians (59.5%) being females. Most of the physicians, including 124 physicians (81%) were practicing in the Eastern Region. Most of the physicians were Saudis, including 129 participants (84.3%), and 78 physicians (51%) were residents. Physicians who had five years or less of experience constitute 78 physicians (51%). The most common specialty was emergency medicine in 52 participants (34%), while the most common hospital type was government hospital in 62 participants (40.5%).

**Table 1:** Physicians' socio-demographic characteristics

Study data	N (%)
Age group	
<30 years	54 (35.3%)
30-39 years	63 (41.2%)
≥40 years	36 (23.6%)
Gender	
Male	62 (40.5%)
Female	91 (59.5%)
Region of practice	
Eastern Region	124 (81.0%)
Western Region	06 (03.9%)
Central Region	17 (11.1%)
Northern Region	03 (02.0%)
Southern Region	03 (02.0%)
Nationality	
Saudi	129 (84.3%)
Non-Saudi	24 (15.7%)
Professional rank	
Resident	78 (51.0%)
Specialist	27 (17.6%)
Consultant	48 (31.4%)
Years of experience	
≤5 years	78 (51.0%)
>5 years	75 (49.0%)
Specialty	
Emergency medicine	52 (34.0%)
Pediatrics	38 (24.8%)
Orthopedics	04 (02.6%)
Family medicine	28 (18.3%)
Others	31 (20.3%)
Type of hospital	
Primary healthcare	21 (13.7%)
Private hospital	12 (07.8%)
Government hospital	62 (40.5%)
University hospital	52 (34.0%)
Military hospital	06 (03.9%)

Regarding physicians' general awareness (Table 2), physicians who asked questions about the possibility of child abuse among patients constituted 100 participants (65.4%). Around 141 physicians (92.2%) agreed that a case of suspected child abuse should be reported, and 93 physicians (60.8%) knew where to refer a child for further evaluation and management of suspected child abuse. The number of physicians who reported a suspected case of child abuse to the authorities in the last six months was 21 physicians (13.7%). Physicians who were aware of the reporting requirement in suspected cases of child abuse were 34 physicians (22.2%). Also, 46 participants (30.1%) indicated that their institution has a written protocol for the management of suspected cases of child abuse. Regarding determinants of awareness, only 26 participants (17%) were correct about the hotline number to report a suspected case of child abuse in Saudi Arabia. Nearly 107 participants (69.9%) were aware of organizations responsible for child protection against abuse. Around 117 physicians (76.5%) knew of their role when encountering a suspected case of child abuse. However, the awareness of how to respond when a family member asked for a medical report of suspected child abuse was poor, as only 35 participants (22.9%) had the correct answer, while 109 participants (71.2%) had the correct response when a family member refused to cooperate about a suspected child abuse encounter. The total mean awareness score was 2.58 (SD 1.01), with poor, moderate, and good awareness levels constituting 65 physicians (42.5%), 62 physicians (40.5%), and 26 physicians (17%), respectively.

**Table 2:** General awareness about child abuse

General awareness	N (%)
Have you ever raised the possibility of child abuse among your patients?	
Yes	100 (65.4%)
No	53 (34.6%)
If you encounter a case of suspected child abuse, are you willing to report it?	
Yes	141 (92.2%)
No	12 (07.8%)
Do you know where to refer the child for further evaluation and management of suspected abuse?	
Yes	93 (60.8%)
No	60 (39.2%)
Have you reported a suspected case of child abuse to the authorities in the last six months?	
Yes	21 (13.7%)
No	132 (86.3%)
Do you know the reporting requirement in suspected cases of child abuse?	
Yes	34 (22.2%)
No	119 (77.8%)
Does your institution have a written protocol for the management of suspected cases of child abuse?	
Yes	46 (30.1%)
No	21 (13.7%)
Not sure	86 (56.2%)
Determinant of awareness toward child abuse	
What is the hotline number to report a suspected case of child abuse in Saudi Arabia? (116111)	26 (17.0%)
Which organizations are responsible for child protection against abuse? (All of the above)	107 (69.9%)
What is your role when encountering a suspected case of child abuse? (Contact the child protection authority)	117 (76.5%)
How do you respond when a family member asks for a medical report of suspected child abuse? (Inform the police and refer the child to a forensic physician for further evaluation)	35 (22.9%)
How do you respond when a family member refuses to cooperate about a suspected child abuse encounter? (Write a report and call the Child Protection Authority to follow up with the case)	109 (71.2%)
Total awareness score (mean ± SD)	2.58 ± 1.01
Level of awareness	
Poor	65 (42.5%)
Moderate	62 (40.5%)
Good	26 (17.0%)

**Figure 1:**
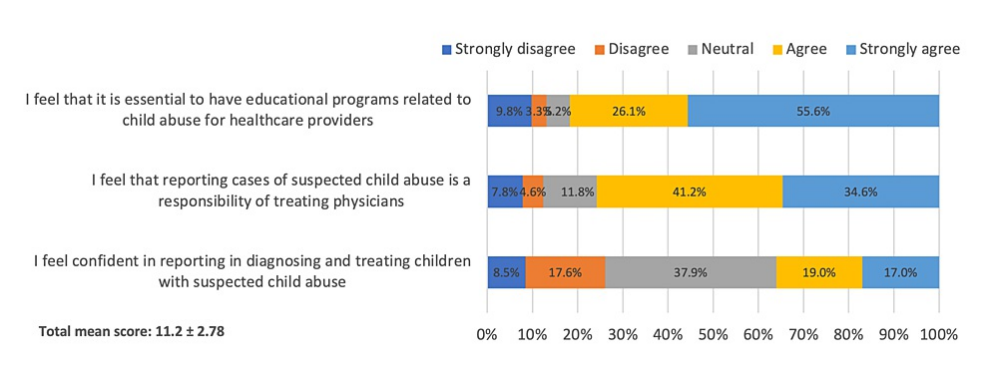
Attitude toward child abuse

**Figure 2:**
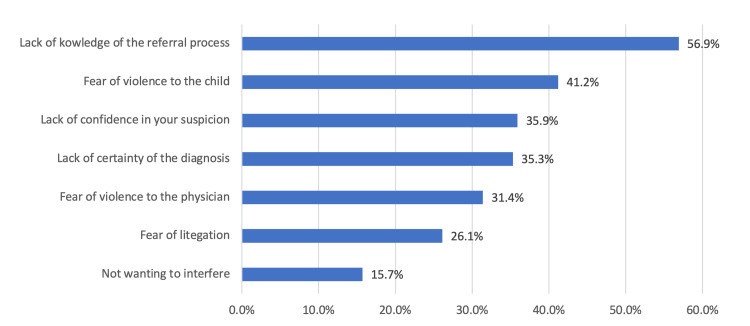
Barrier to refer child with abuse

Regarding the attitude toward child abuse (Figure 1), 85 participants (55.6%) strongly agreed that it is essential to have educational programs related to child abuse for healthcare providers. Also, 53 participants (34.6%) felt that reporting cases of suspected child abuse is the responsibility of treating physicians, and 26 participants (17%) strongly agreed with having confidence in reporting, diagnosing, and treating children with suspected child abuse. The overall mean attitude score was 11.2 (SD 2.78). In Figure 2, the most common barrier to referring a child with abuse was the lack of knowledge of the referral process in 87 physicians (56.9%), followed by fear of violence to the child in 63 physicians (41.2%), and lack of confidence in your suspicion in 55 physicians (35.9%).

In the assessment of knowledge in recognizing child abuse based on radiographs of different cases (Table 3), our results showed that physicians demonstrated good knowledge, particularly in cases 2, 3, and 4. However, poor knowledge was seen in other cases of child abuse as correct responses were unsatisfactory, most notably in case 10: evaluating a two-year-old child with bruises for suspected abuse in 50 physicians (32.7%), case 6: a 12-month-old infant presenting to the ED for scalp swelling in 44 physicians (28.8%), case 11: evaluation of multiple patients with different clinical presentation during on-call hours in 26 physicians (17%), and case 8: evaluation of burns on a three-year-old brought to the emergency department in 12 physicians (7.8%). Based on the correct answer for all cases, the overall mean knowledge score was 7.07 (SD 3.54), with poor, moderate, and good knowledge levels constituting 69 physicians (45.1%), 79 physicians (51.6%), and 5 physicians (3.3%), respectively.

**Table 3:** Assessment of knowledge in recognizing physical child abuse based on radiographs of different case scenarios

Case items	N (%)
Case 1: A two-year-old child was brought to the emergency department with weakness and poor growth.	
Is there a fracture? (no)	90 (58.8%)
Is the fracture highly suspicious of child abuse? (no)	08 (19.0%)
Case 2: A seven-day-old child had excessive, inconsolable crying after his mother tripped and landed on him.	
Is there a fracture? (yes)	114 (74.5%)
Is the fracture highly suspicious of child abuse? (yes)	94 (61.4%)
Case 3: A one-year-old child was brought to the emergency department with multiple bruises.	
Is there a fracture? (yes)	112 (73.2%)
Is the fracture highly suspicious of child abuse? (yes)	109 (71.2%)
Case 4: A four-year-old child was brought to the emergency department with leg swelling after he jumped off the bed.	
Is there a fracture? (yes)	121 (79.1%)
Is the fracture highly suspicious of child abuse? (yes)	62 (40.5%)
Case 5: A four-year-old child was brought to the emergency department with knee pain.	
Is there a fracture? (no)	74 (48.4%)
Is the fracture highly suspicious of child abuse? (no)	27 (17.6%)
Case 6: A 12-month-old infant presented to the ED for scalp swelling.	
Which of the following is the least suggestive of non-accidental skull fracture? (parietal bone fracture)	44 (28.8%)
Case 7: A 12-month-old infant underwent a skeletal survey.	70 (45.8%)
Which of the following fractures is least concerning for child abuse? (Humeral fracture with history of fall from more than four feet)	70 (45.8%)
Case 8: A three-year-old girl is brought to the emergency department for evaluation of burns.	
Which of the following features are most concerning for child abuse? (non-uniformity of burn depth)	12 (07.8%)
Case 9: A two-year-old boy was noted to have bruises.	
Which of the following features is least concerning for child abuse? (bruises on site of bony prominence)	69 (45.1%)
Case 10: You are evaluating a two-year-old child with bruises for suspected non-accidental injury	
Which of the following sites is least concerning for child abuse? (shins)	50 (32.7%)
Case 11: During your on-call hours, you evaluated multiple patients with different clinical presentations	
Which of the following cases is most concerning for child abuse? (a six-month-old boy with a torn frenulum)	26 (17.0%)
Total knowledge score (mean ± SD)	7.07 ± 3.54
Level of knowledge	
Poor	69 (45.1%)
Moderate	79 (51.6%)
Good	05 (03.3%)

**Figure 3:**
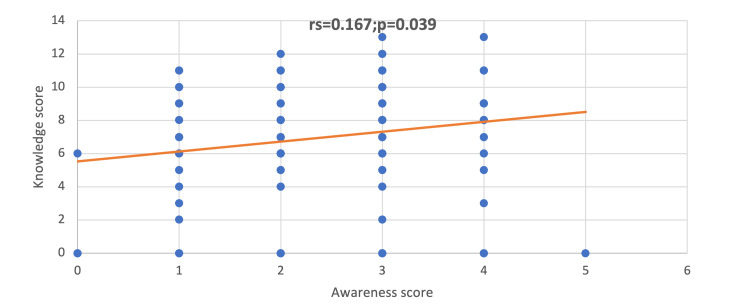
Correlation between the score of awareness and knowledge

**Figure 4:**
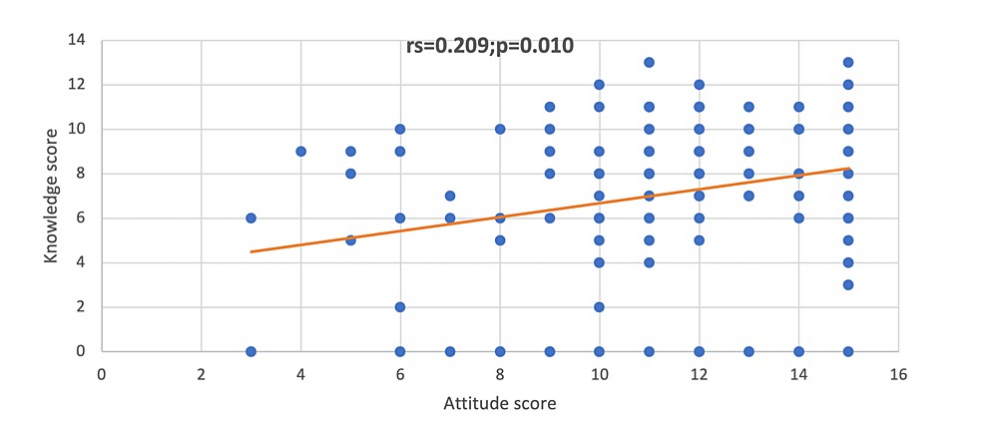
Correlation between the score of knowledge and attitude

Figure 3 shows a positive significant correlation between awareness and knowledge scores (rs = 0.167; p = 0.039), suggesting that the increase in awareness is correlated with the increase in knowledge. On the other hand, Figure 4 shows a positive significant correlation between the knowledge and attitude scores (rs = 0.209; p = 0.010), indicating that the increase in the knowledge score is correlated with the score of attitudes.

When measuring the differences in the score of awareness, attitude, and knowledge in relation to the socio-demographic characteristics of physicians (Table 4), it was found that a higher attitude score was more associated with male physicians (Z = 2.053; p = 0.040), practicing inside Eastern Region (Z = 2.259; p = 0.024), having five years or less experience (Z = 2.422; p = 0.015), having emergency medicine specialty (H = 11.251; p = 0.010) and working in the government/military hospital (H = 9.969; p = 0.019). Also, a higher knowledge score was more associated with physicians practicing inside the Eastern Region (Z = 3.997; p < 0.001), physicians with emergency medicine specialty (H = 19.264; p < 0.001), and physicians working in the government/military hospital (H = 13.718; p = 0.003). However, we found no significant differences in the awareness score in relation to all demographic data (p > 0.05).

**Table 4:** Differences in the score of awareness, attitude, and knowledge in relation to the physicians' socio-demographic characteristics ^§^ P-value has been calculated using Mann Whitney Z-test. ^‡^ P-value has been calculated using the Kruskal Wallis H-test. ^**^ Significant at p < 0.05 level.

Factor	Awareness score (5) mean ± SD	Attitude score (15) mean ± SD	Knowledge score (16) mean ± SD
Age group			
<30 years	2.59 ± 0.86	11.9 ± 2.20	7.00 ± 3.42
30-39 years	2.54 ± 0.96	11.1 ± 2.54	7.54 ± 3.31
≥40 years	2.61 ± 1.29	10.5 ± 3.71	6.36 ± 4.04
H-test; p-value^ ‡^	0.644; 0.725	2.469; 0.291	1.665; 0.435
Gender			
Male	2.59 ± 0.98	11.8 ± 2.68	7.09 ± 3.29
Female	2.56 ± 1.04	10.8 ± 2.79	7.05 ± 3.72
Z-test; p-value ^§^	0.301; 0.764	2.053; 0.040^**^	0.262; 0.793
Region of practice			
Inside Eastern Region	2.63 ± 0.98	11.5 ± 2.51	7.63 ± 3.26
Outside Eastern Region	2.34 ± 1.14	10.0 ± 3.55	4.69 ± 3.75
Z-test; p-value^ §^	1.220; 0.223	2.259; 0.024^**^	3.997; <0.001^**^
Nationality			
Saudi	2.57 ± 0.98	11.4 ± 2.59	7.37 ± 3.48
Non-Saudi	2.58 ± 1.18	10.3 ± 3.58	5.46 ± 3.50
Z-test; p-value^ §^	0.237; 0.813	1.251; 0.211	2.744; 0.006^**^
Professional rank ^‡^			
Trainee resident	2.63 ± 0.94	11.9 ± 2.05	7.12 ± 3.25
Service resident	2.19 ± 1.03	10.4 ± 2.84	5.81 ± 3.72
Specialist	2.37 ± 1.08	10.7 ± 3.34	6.96 ± 3.78
Consultant	2.79 ± 1.01	11.2 ± 3.08	7.63 ± 3.62
H-test; p-value	5.987; 0.112	4.744; 0.192	4.338; 0.227
Years of experience			
≤5 years	2.55 ± 0.95	11.9 ± 2.19	7.06 ± 3.46
>5 years	2.60 ± 1.08	10.5 ± 3.15	7.08 ± 3.64
Z-test; p-value ^§^	0.560; 0.576	2.422; 0.015^**^	0.132; 0.895
Specialty ^‡^			
Emergency medicine	2.54 ± 0.87	11.9 ± 2.43	8.48 ± 3.06
Pediatrics	2.66 ± 1.21	11.6 ± 2.35	7.08 ± 3.57
Family Medicine	2.61 ± 1.03	9.68 ± 3.29	5.54 ± 3.42
Others	2.51 ± 0.98	11.1 ± 2.88	6.20 ± 3.61
H-test; p-value	1.025; 0.795	11.251; 0.010^**^	19.264; <0.001^**^
Type of hospital ^‡^			
Primary healthcare	2.52 ± 1.08	9.57 ± 2.79	5.71 ± 3.13
Private hospital	2.25 ± 1.29	10.6 ± 3.65	4.25 ± 4.07
Government/military hospital	2.60 ± 1.01	11.6 ± 2.59	7.81 ± 2.77
University hospital	2.63 ± 0.94	11.5 ± 2.60	7.39 ± 2.77
H-test; p-value	1.165; 0.761	9.969; 0.019^**^	13.718; 0.003^**^

## Discussion

The measurement of physicians’ awareness, knowledge, and attitude toward child abuse, reflects the first liners’ capability of detecting it and preventing further damage. The Centers for Disease Control and Prevention (CDC) defines child abuse/maltreatment as "any act or series of acts of commission or omission by a parent or other caregiver that results in harm, potential for harm, or threat of harm to a child. Acts of omission include physical abuse, sexual abuse, and psychological abuse, and acts of omission include neglect (physical, emotional, medical/dental, or educational) and failure to supervise" [1]. Furthermore, the Child Abuse Prevention and Treatment Act (CAPTA) defines it as "a recent act or failure to act that results in death, serious physical or emotional harm, sexual abuse or exploitation, or imminent risk of serious harm; involves a child; and is carried out by a parent or caregiver who is responsible for the child's welfare" [11].

These definitions display two major pillars of child abuse, the concept of behavior and consequences that involve children as victims. The concept of behavior is either a performed act of intended or consequent possible harm (commission), which can be in different forms (e.g., physical trauma, sexual abuse) or refraining from performing basic requirements that maintain the child’s health or possibly harm them if not met (omission), which can be in several forms (e.g., nutritional, or psychological neglect). On the other hand, the consequences can manifest in either physical (e.g., injured body parts or death), physiological (e.g., substance use disorders), or societal (e.g., mass shootings) debilitating effects [12-15]. Hence, the clinical presentation is form-dependent (i.e., physical trauma will manifest in several bruises of different healing phases, long bone fractures, or circumscribed burns, while psychological abuse is subtle and may present later in life in the form of chronic disorders, mood disorders or decreased academic performance), in which lays the difficulty in detection [16,17].

The general awareness of the topic in our sample was poor, moderate, and good awareness levels constituting 65 physicians (42.5%), 62 physicians (40.5%), and 26 physicians (17%), respectively. In addition, 141 participants (92.2%) of the sample agreed that if a child abuse case is encountered, it should be reported, while 93 participants (60.8%) knew the steps to take for further management. These findings display that despite the majority of the sample having poor-moderate levels of awareness, they are willing to report and further manage these cases. The willingness to report these cases was depicted in more than half of the sample’s attitude, including 85 physicians (55.6%) who strongly agree on the essentiality of having training programs for physicians on this topic, and 53 physicians (34.6%) felt that the reporting process is primarily the physician’s task. In addition, the knowledge assessment results were poor in 69 physicians (45.1%), moderate in 79 physicians (51.6%), and good in 5 physicians (3.3%) levels, which are comparable to Alaraik et al.’s study results where their overall correct answers rate was approximately in half of the specimen’s answers (53%) and 47% having an insufficient level of knowledge [18]. Furthermore, the relation between elevated levels of knowledge and sociodemographics was established in our study with being an emergency medicine physician working in a governmental/military hospital, in the eastern province, which can be secondary to the level of patients’ exposure and level of experience. Therefore, institution-based training programs (i.e., programs that break down the multifactorial nature of child abuse, clinical stigmata, and methods of management) and public campaigns are an integral part of maintaining our children's future health [19].

The absence of proper training was reflected in the barriers to reporting suspected victims with the top reasons being a lack of knowledge of the referral process in 87 physicians (56.9%), fear of violence to the child in 63 physicians (41.2%), and lack of confidence in your suspicion in 55 physicians (35.9%). Alkathiri et al. had similar results with the lack of knowledge about reporting procedures (56.8%) being the most common, and uncertainty about the diagnosis in 41.9% [20]. Moreover, Alsaleem et al. reported that the most common reason was an unclear reporting procedure (43.7%) [21]. Accordingly, a nationwide, clear program should be established to enable physicians to aid the victims (i.e., a program that clarifies the clinical pathway of any suspected patient, ensuring that they receive proper care), empower physicians (i.e., state their concerns in confidentiality), and sustain the victims’ safety. The expected benefits of training programs were anticipated by an established correlation in our results, in which an increment in awareness increases knowledge (p = 0.039) and knowledge subsequently increases the physician’s positive attitude (p = 0.010) (i.e., their capability to handle the external circumstances of these incidents). These findings can be reflected in our medical education infrastructure to bridge this gap in knowledge and improve our children’s outcomes [22].

**Figure 5:**
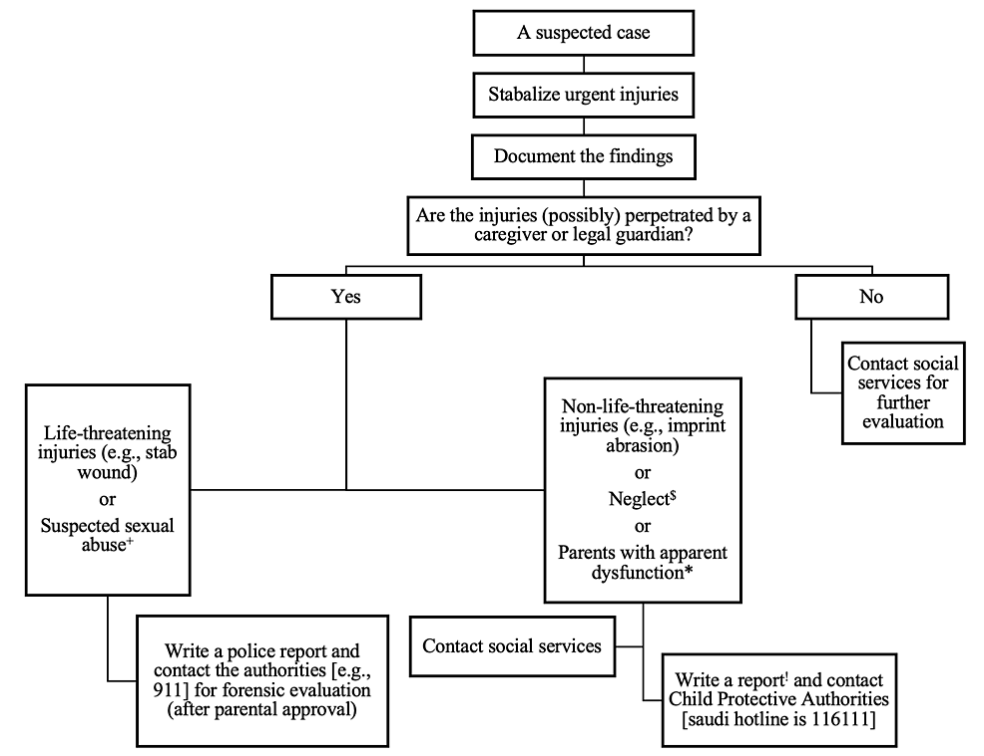
An approach to suspected cases of child abuse Note: Authors' own creation. ^+^ Cases with symptomology of (e.g., sexually transmitted diseases, genital abrasions or lesions, fearful reaction to a caregiver, speaks about acts of normalized exhibitionism). ^*^ Apparent dysfunction is a behavioral or psychological issue that deems a caregiver possibly harmful to themselves or their children (e.g., a parent with psychotic manifestations, substance use disorder, bed-ridden “if a single parent,” neurological deficits in memory/disinhibition, or sustained disabling injuries to take care of the child). ^$^ Nutritional neglect can be life-threatening in the form of severe dehydration sequelae (e.g., seizure); however, ruling out possible differentials is important prior to contacting other services (e.g., avoidant/restrictive food intake disorder). ^!^ Using a specific reporting form (social services can help with the format).

In an attempt to bridge the gap, Figure 5 was formulated to display a possible approach to manage suspected cases of child abuse, by combining the Saudi Arabian law and experts’ opinions in our institute [23]. Accordingly, admission could be advised for further evaluation of suspected cases; however, separation of the child from the legal guardians is not allowed until harm is proven (by law) to be inflicted by the guardians. In addition, in some cases, warning-inflicted injuries can be seen (e.g., bruises or abrasion over the back and buttocks) in which admission is favorable for evaluation, yet the diagnosis of child abuse/neglect should be considered after ruling out possible congenital or acquired mimickers (e.g., hemophilia, erythema migrans) [24]. Furthermore, there’s an institute-based form (approved by the Ministry of Saudi Arabian Health) to report the cases in direct and plain language.

Limitations and recommendations

The study’s design is cross-sectional, reflecting only a point in time for physicians from different specialties’ knowledge about the topic. In addition, it’s questionnaire-based which can involve technical (e.g., submitting half of the questions answered), question-related (e.g., an unclear image on the phone), or physician-related (e.g., did not revise the topic recently). Therefore, we urge future studies to perform an assessment of physicians’ awareness about the topic before and after a training workshop and then after a period of time (e.g., three months), blindly (i.e., sent without informing the participants priorly). In addition, incorporating this topic into the medical education programs to increase the awareness of future physicians on this phenomenon.

## Conclusions

In conclusion, it was shown that a significant number of physicians had poor awareness and moderate knowledge of child abuse. Also, lack of knowledge was the most common barrier for physicians to report child abuse. Thus, this study highlights the importance of implementing specialized training programs and workshops focused on identifying and reporting child abuse, as well as providing resources and guidelines for recognizing signs of abuse and taking appropriate intervention measures which will increase the knowledge, awareness, and attitude of physicians toward child abuse. To gain a better understanding of this topic, future studies should be conducted using a larger sample size and involving physicians from multiple countries.
